# *R*-Norm Entropy and *R*-Norm Divergence in Fuzzy Probability Spaces

**DOI:** 10.3390/e20040272

**Published:** 2018-04-11

**Authors:** Dagmar Markechová, Batool Mosapour, Abolfazl Ebrahimzadeh

**Affiliations:** 1 Department of Mathematics, Faculty of Natural Sciences, Constantine The Philosopher University in Nitra, A. Hlinku 1, Nitra SK-949 01, Slovakia; 2 Department of Mathematics, Farhangian University, Kerman +98-7616914111, Iran; 3 Young Researchers and Elite Club, Zahedan Branch, Islamic Azad University, Zahedan +98-9816883673, Iran

**Keywords:** fuzzy measurable space, fuzzy P-measure, fuzzy probability space, fuzzy partition, *R*-norm entropy, conditional *R*-norm entropy, *R*-norm divergence

## Abstract

In the presented article, we define the *R*-norm entropy and the conditional *R*-norm entropy of partitions of a given fuzzy probability space and study the properties of the suggested entropy measures. In addition, we introduce the concept of *R*-norm divergence of fuzzy P-measures and we derive fundamental properties of this quantity. Specifically, it is shown that the Shannon entropy and the conditional Shannon entropy of fuzzy partitions can be derived from the *R*-norm entropy and conditional *R*-norm entropy of fuzzy partitions, respectively, as the limiting cases for *R* going to 1; the Kullback–Leibler divergence of fuzzy P-measures may be inferred from the *R*-norm divergence of fuzzy P-measures as the limiting case for *R* going to 1. We also provide numerical examples that illustrate the results.

## 1. Introduction

The concept of information entropy was introduced by Claude Shannon in 1948 in his article [1]. It is used in information theory [2] to quantify the amount of information or uncertainty inherent in a system. We remind that Shannon’s entropy is defined in the context of a probabilistic model. Consider a measurable partition $A=\left\{E_{1},E_{2},\ldots ,E_{n}\right\}$ of a probability space (X,S,P) (that is a finite collection of measurable subsets $E_{k}\subset X,$ k=1,2,…,n, such that $\cup_{k=1}^{n}E_{k}=X,$ and $E_{i}\cap E_{j}=\emptyset$ whenever i≠j) with probabilities $p_{k}=P(E_{k}),$ k=1,2,…,n. Then the Shannon entropy of A is defined as the number $h(A)=-\sum_{k=1}^{n}p_{k}\cdot \log_{b}p_{k}$ with the convention that $0\cdot \log_{b}0=0$ (which is justified by the fact that $\underset{p\to 0}{\lim}p\cdot \log_{b}\frac{1}{p}=0$). The base of the logarithm can be any positive real number; depending on the selected base of the logarithm, the entropy is expressed in bits (b=2), nats (b=e), or dits (b=10).

The extensions of Shannon’s entropy have led to several alternatives of entropy measure, of which the Rényi entropy [3] is one of the most important. The classical logical entropy (cf. [4,5]) and the entropy measure called the *R*-norm entropy (cf. [6,7]) are other alternative entropy measures. In this article, we will deal with the study of the *R*-norm entropy. If $P=\left\{p_{1},p_{2},\ldots ,p_{n}\right\}$ is a probability distribution, then the *R*-norm entropy is defined, for every real number R∈(0,1)∪(1,∞), by the formula:

$$H_{R}(P)=\frac{R}{R-1}\left(1-{\left[\sum_{k=1}^{n}p_{k}^{R}\right]}^{\frac{1}{R}}\right).$$

Some results regarding the *R*-norm entropy measure and its generalizations can be found in [8,9,10,11,12]. The above entropy measures have found many important applications, for example, in statistics, pattern recognition, and coding theory.

In classical probability theory, partitions are defined in the context of the Cantor set theory. In solving many real-life problems, however, the partitions defined in terms of fuzzy set theory [13] are more appropriate. Therefore, many proposals have been made to generalize the classical partitions into fuzzy partitions [14,15,16,17,18,19,20]. Fuzzy partitions represent a mathematical tool for modeling random experiments that lead to unclear, vague events. Naturally, there are also many results concerning the Shannon entropy of fuzzy partitions; see e.g., [21,22,23,24,25,26,27,28,29,30,31]. We note that in [32] the results regarding the entropy of fuzzy partitions provided in [25] have been employed to introduce the notions of mutual information and Kullback–Leibler divergence for the fuzzy case. The notion of Kullback–Leibler divergence was introduced in [33] as the distance measure between two probability distributions. It plays significant roles in information theory and various disciplines such as statistics, machine learning, physics, neuroscience, computer science, linguistics, etc.

Since its inception in 1965, the theory of fuzzy sets has advanced in many mathematical disciplines and has found important applications in practice. Currently, the subjects of intense study are algebraic systems based on the theory of fuzzy sets, for example, D-posets [34,35,36], MV-algebras [37,38,39,40,41], and effect algebras [42]. Some results regarding the above entropy measures and divergence on these structures can be found e.g., in [43,44,45,46,47,48,49,50,51,52,53,54,55,56,57,58].

The aim of this article is to study the *R*-norm entropy of fuzzy partitions and the *R*-norm divergence in fuzzy probability spaces [59]. The organization of the paper is as follows. In Section 2 we provide basic definitions, terminology and some known results used in the paper. The results of the article are presented in Section 3 and Section 4. In Section 3, we define the *R*-norm entropy and conditional *R*-norm entropy of fuzzy partitions and examine their properties. In Section 4, the concept of the *R*-norm divergence for the case of fuzzy probability spaces is proposed and the properties of this distance measure are studied. The results presented in Section 3 and Section 4 are illustrated with numerical examples. The paper concludes in Section 5 with a brief summary.

## 2. Preliminaries

We begin by recalling the basic concepts and the known results used in the paper.

It is known that the concept of fuzzy set, introduced by Zadeh in 1965 [13], extends the classical set theory. In classical set theory, the membership of elements in a set is assessed in binary terms according to the condition—an element either belongs or does not belong to the considered set. By contrast, the fuzzy set is characterized by a membership function which assigns to every element a grade of membership ranging between zero and one. The mathematical model of the fuzzy set is as follows. Let *X* be a non-empty set. By a fuzzy subset of *X* we mean a mapping a:X→[0,1] (where the considered fuzzy set is identified with its membership function). The value a(x) is interpreted as a grade of membership of the element x∈X to the considered fuzzy set a.

**Definition** **1.** *Let X be a non-empty set, and* $M\subset {\left[0,1\right]}^{X}$ *be a family of fuzzy subsets of X. The pair* (X,M) *is called a fuzzy measurable space, if the following conditions are satisfied: (i)* $1_{X}\in M,{\left(1/2\right)}_{X}\notin M;$ *(ii)* $a\in M\Rightarrow a^{⊥}\in M;$ *(iii) if* $a_{n}\in M,n=1,2,\ldots ,$ *then* $\cup_{n=1}^{\infty }a_{n}\in M.$ *The family* $M\subset {\left[0,1\right]}^{X}$ *with the properties (i)*–*(iii) is said to be a fuzzy* σ-*algebra.*

Throughout the paper, the symbols $\cup_{n=1}^{\infty }a_{n}$ and $\cap_{n=1}^{\infty }a_{n}$ denote the fuzzy union and the fuzzy intersection of a sequence ${\left\{a_{n}\right\}}_{n=1}^{\infty }\subset M,$ respectively, by Zadeh [13], i.e., $\cup_{n=1}^{\infty }a_{n}=\sup_{n}a_{n},$ and $\cap_{n=1}^{\infty }a_{n}=\inf_{n}a_{n}.$ The symbol $a^{⊥}$ denotes the complement of fuzzy set a∈M, i.e., $a^{⊥}=1_{X}-a.$ Here, $1_{X}$ denotes the constant function with the value 1; analogously, the symbols ${\left(1/2\right)}_{X}$ and $0_{X}$ denote the constant functions with the value 1/2, and 0, respectively. Additionally, the relation ≤ denotes the usual order relation of fuzzy subsets of *X*, i.e., a≤b if and only if a(x)≤b(x), for every x∈X. The complementation $⊥:a\to a^{⊥},$ a∈M, satisfies, for every a,b∈M, the conditions: (i) ${\left(a^{⊥}\right)}^{⊥}=a,$ and (ii) a≤b implies $b^{⊥}\leq a^{⊥}.$

Fuzzy subsets a,b∈M with the property $a\cap b=0_{X}$ are said to be separated, fuzzy subsets a,b∈M with the property $a\leq b^{⊥}$ are said to be W-separated fuzzy sets. Any fuzzy subset a∈M with the property $a\geq a^{⊥}$ is said to be a W-universum, any fuzzy subset a∈M with the property $a\leq a^{⊥}$ is said to be a W-empty fuzzy set. A fuzzy set from the fuzzy σ-algebra *M* is interpreted as a fuzzy event. W-separated fuzzy events are considered to be mutually exclusive events. A W-universum is interpreted as a certain event, a W-empty set as an impossible event. It can be shown that a fuzzy subset a∈M is a W-universum if and only if there exists a fuzzy subset b∈M such that $a=b\cup b^{⊥}.$

Naturally, the notion of a fuzzy measurable space generalizes the concept of a measurable space (X,S) from the classical measure theory; it suffices to put $M=\left\{\chi_{E}:E\in S\right\},$ where $\chi_{E}$ is the characteristic function of the set E∈S. With this procedure, the classical model can be inserted into the fuzzy one.

**Definition** **2** ([59])**.** *Let* (X,M) *be a fuzzy measurable space. A map* μ:M→[0,1] *is said to be a fuzzy P-measure, the following conditions being satisfied: (i)* $\mu (a\cup a^{⊥})=1,$ *for every* a∈M; *(ii) if* ${\left\{a_{n}\right\}}_{n=1}^{\infty }$ *is a sequence of pairwise W-separated fuzzy sets from M, then* $\mu \left(\cup_{n=1}^{\infty }a_{n}\right)=\sum_{n=1}^{\infty }\mu \left(a_{n}\right).$ *The triplet* (X,M,μ) *is called a fuzzy probability space.*

The fuzzy P-measure μ:M→[0,1] has the properties that correspond to properties of a classical probability measure; the proofs can be found in [59].

(P1) $\mu (a^{⊥})=1-\mu (a),$ for every a∈M.
(P2) μ is non-decreasing, i.e., if a,b∈M with a≤b, then μ(a)≤μ(b).
(P3) μ(a∪b)+μ(a∩b)=μ(a)+μ(b), for every a,b∈M.
(P4) Let b∈M. Then μ(a∩b)=μ(a) for all a∈M if and only if μ(b)=1.
(P5) If a,b∈M such that $a\leq b^{⊥},$ then μ(a∩b)=0.
(P6) If a,b∈M such that a≤b, then $\mu (b)=\mu (a)+\mu (a^{⊥}\cap b).$

**Definition** **3** ([14])**.** *A fuzzy partition of a fuzzy probability space* (X,M,μ) *is a collection* $A=\left\{a_{1},a_{2},\ldots ,a_{n}\right\}$ *of W-separated fuzzy sets from M with the property* $\mu \left(\cup_{i=1}^{n}a_{i}\right)=1.$

In the system of all fuzzy partitions of (X,M,μ), we define the refinement partial order in the following way. If *A* and *B* are two fuzzy partitions of (X,M,μ), then we say that *B* is a refinement of *A* (and write A≺B), if for every b∈B there exists a∈A such that b≤a. Furthermore, for every two fuzzy partitions $A=\left\{a_{1},a_{2},\ldots ,a_{n}\right\},$ and $B=\left\{b_{1},b_{2},\ldots ,b_{m}\right\}$ of (X,M,μ), we put A∨B= $\left\{a_{i}\cap b_{j};i=1,2,\ldots ,n,j=1,2,\ldots ,m\right\}.$ One can easily to verify that the family A∨B is a family of pairwise W-separated fuzzy sets from *M*; moreover, by the property (P4), we have $\mu \left(\cup_{i=1}^{n}\cup_{j=1}^{m}\left(a_{i}\cap b_{j}\right)\right)=\mu \left(\left(\cup_{i=1}^{n}a_{i}\right)\cap \left(\cup_{j=1}^{m}b_{j}\right)\right)=\mu \left(\cup_{i=1}^{n}a_{i}\right)=1.$ Thus, A∨B is a fuzzy partition of (X,M,μ). It represents a combined experiment consisting of a realization of the experiments *A* and *B*. Evidently, it holds A≺A∨B, and B≺A∨B, i.e., the fuzzy partition A∨B is a common refinement of fuzzy partitions *A* and *B*. If $A_{1},A_{2},\ldots ,A_{n}$ are fuzzy partitions of (X,M,μ), then we put $\vee_{i=1}^{n}A_{i}=A_{1}\vee A_{2}\vee \ldots \vee A_{n}.$

**Definition** **4.** *Two fuzzy partitions* $A=\left\{a_{1},a_{2},\ldots ,a_{n}\right\},$ *and* $B=\left\{b_{1},b_{2},\ldots ,b_{m}\right\}$ *of a fuzzy probability space* (X,M,μ) *are said to be statistically independent, if* $\mu (a_{i}\cap b_{j})=\mu (a_{i})\cdot \mu (b_{j}),$ *for* i=1,2,…,n, j=1,2,…,m.

**Example** **1.** *Let us consider a classical probability space* (X,S,P), *and put* $M=\left\{\chi_{E}:E\in S\right\}.$ *It can be verified that the map* μ:M→[0,1] *defined by* $\mu (\chi_{E})=P(E),$ *for every* $\chi_{E}\in M,$ *is a fuzzy P-measure and the triplet* (X,M,μ) *is a fuzzy probability space. A classical measurable partition* $A=\left\{E_{1},E_{2},\ldots ,E_{n}\right\}$ *of a probability space* (X,S,P) *can be eventually regarded as a fuzzy partition of* (X,M,μ), *considering* $\chi_{E_{i}}$ *instead of* $E_{i}.$

The Shannon entropy of fuzzy partition of a fuzzy probability space (X,M,μ) has been introduced and examined in [23], see also [25].

**Definition** **5** ([23])**.** *We define the entropy of a fuzzy partition* $A=\left\{a_{1},a_{2},\ldots ,a_{n}\right\}$ *of* (X,M,μ) *by Shannon’s formula:*

(1) $$H^{\mu }(A)=-\sum_{i=1}^{n}\mu (a_{i})\cdot \log\mu (a_{i}).$$

*If* $A=\left\{a_{1},a_{2},\ldots ,a_{n}\right\},$ *and* $B=\left\{b_{1},b_{2},\ldots ,b_{m}\right\}$ *are two fuzzy partitions of* (X,M,μ), *then we define the conditional entropy of A given B by the formula:*

(2) $$H^{\mu }(A/B)=-\sum_{i=1}^{n}\sum_{j=1}^{m}\mu (a_{i}\cap b_{j})\cdot \log\frac{\mu (a_{i}\cap b_{j})}{\mu (b_{j})}$$

*with the convention that* $0\cdot \log\frac{0}{x}=0$ *if* x≥0.

The symbol log denotes the base 2 logarithm, so the Shannon entropy of fuzzy partition is expressed in bits. The entropy and the conditional entropy of fuzzy partitions have properties that correspond to properties of Shannon’s entropy of classical measurable partitions: for every fuzzy partitions A,B,C of a fuzzy probability space (X,M,μ), it holds:

(S1) A≺B implies $H^{\mu }(A)\leq H^{\mu }(B);$
(S2) $H^{\mu }(A\vee B/C)=H^{\mu }(A/C)+H^{\mu }(B/C\vee A);$
(S3) A≺B implies $H^{\mu }(A/C)\leq H^{\mu }(B/C);$
(S4) A≺B implies $H^{\mu }(C/A)\geq H^{\mu }(C/B);$
(S5) $H^{\mu }(A/B)\leq H^{\mu }(A)$ with the equality if and only if A,B are statistically independent;
(S6) $H^{\mu }(B\vee C/A)\leq H^{\mu }(B/A)+H^{\mu }(C/A);$
(S7) $H^{\mu }(A\vee B)=H^{\mu }(A)+H^{\mu }(B/A).$

The proofs can be found in [23,25]. We remark that in [15,16,17,18,19,20,21,22,26,27,28,29,30,31], other conceptions of fuzzy partitions and their entropy measures have been introduced. Whereas our approach is based on Zadeh’s connectives, in the referenced papers Zadeh’s connectives have been replaced by other fuzzy set operations.

We note that in [32], the concept of Kullback–Leibler divergence in the fuzzy probability space was introduced. Let μ, υ be two fuzzy P-measures on a fuzzy measurable space (X,M), and $A=\left\{a_{1},a_{2},\ldots ,a_{n}\right\}$ be a fuzzy partition of fuzzy probability spaces (X,M,μ), (X,M,υ). Then the Kullback–Leibler divergence of fuzzy P-measures μ, υ with respect to A is defined as the number:

(3) $$D_{A}(\mu ∥\upsilon )=\sum_{i=1}^{n}\mu (a_{i})\cdot \log\frac{\mu (a_{i})}{\upsilon (a_{i})}$$

with the convention that $x\log\frac{x}{0}=\infty$ if x>0, and $0\cdot \log\frac{0}{x}=0$ if x≥0.

In the following sections, we will use the following known Minkowski inequality: for non-negative real numbers $x_{1},x_{2},\ldots ,x_{n},y_{1},y_{2},\ldots ,y_{n},$ it holds:

$${\left[\sum_{i=1}^{n}{x_{i}}^{R}\right]}^{\frac{1}{R}}+{\left[\sum_{i=1}^{n}{y_{i}}^{R}\right]}^{\frac{1}{R}}\geq {\left[\sum_{i=1}^{n}{\left(x_{i}+y_{i}\right)}^{R}\right]}^{\frac{1}{R}},\;\mathrm{for}\;R>1,$$

and

$${\left[\sum_{i=1}^{n}{x_{i}}^{R}\right]}^{\frac{1}{R}}+{\left[\sum_{i=1}^{n}{y_{i}}^{R}\right]}^{\frac{1}{R}}\leq {\left[\sum_{i=1}^{n}{\left(x_{i}+y_{i}\right)}^{R}\right]}^{\frac{1}{R}},\;\mathrm{for}\;0<R<1.$$

Furthermore, we will use the Jensen inequality which states that for a real convex function φ, real numbers $x_{1},x_{2},\ldots ,x_{m}$ in its domain and non-negative real numbers $\alpha_{1},\alpha_{2},\ldots ,\alpha_{m}$ with $\sum_{j=1}^{m}\alpha_{j}=1,$ it holds:

$$\varphi \left(\sum_{j=1}^{m}\alpha_{j}x_{j}\right)\leq \sum_{j=1}^{m}\alpha_{j}\varphi (x_{j}),$$

and the inequality is reversed if φ is a real concave function. The equality holds if and only if $x_{1}=x_{2}=\ldots =x_{m}$ or φ is linear.

In addition, we will use L’Hôpital’s rule: for functions f and g that are differentiable on an open interval *U* except possibly at a point a∈U, if $\underset{x\to a}{\lim}f(x)=$$\underset{x\to a}{\lim}g(x)=0,$ $g^{\prime }(x)\neq 0,$ for every *x* in U with x≠a, and $\underset{x\to a}{\lim}\frac{f^{\prime }(x)}{g^{\prime }(x)}$ exists, then:

$$\underset{x\to a}{\lim}\frac{f(x)}{g(x)}=\underset{x\to a}{\lim}\frac{f^{\prime }(x)}{g^{\prime }(x)}.$$

## 3. The *R*-Norm Entropy of Fuzzy Partitions

In this part we define the *R*-norm entropy of a fuzzy partition and its conditional version and study the properties of these entropy measures. It is shown that as the limiting cases of the *R*-norm entropy and the conditional *R*-norm entropy of fuzzy partitions for *R* going to 1, we obtain the Shannon entropy $H^{\mu }(A),$ and the conditional Shannon entropy $H^{\mu }(A/B),$ respectively, expressed in nats.

**Definition** **6.** *Let* $A=\left\{a_{1},a_{2},\ldots ,a_{n}\right\}$ *be a fuzzy partition of a fuzzy probability space* (X,M,μ). *The R-norm entropy of A with respect to* μ *is defined, for a positive real number R not equal to* 1*, by the formula:*

(4) $$H_{R}^{\mu }(A)=\frac{R}{R-1}\left(1-{\left[\sum_{i=1}^{n}\mu {\left(a_{i}\right)}^{R}\right]}^{\frac{1}{R}}\right).$$

**Remark** **1.** *For simplicity, we write* $\mu {\left(a_{i}\right)}^{R}$ *instead of* ${\left(\mu (a_{i})\right)}^{R}.$ *In the following, we will write* $\log\sum_{i=1}^{n}\mu {\left(a_{i}\right)}^{R}$ *instead of* $\log\left(\sum_{i=1}^{n}\mu {\left(a_{i}\right)}^{R}\right).$

**Theorem** **1.** *For arbitrary fuzzy partition* A *of a fuzzy probability space* (X,M,μ), *the R-norm entropy* $H_{R}^{\mu }(A)$ *is non-negative.*

**Proof.**  Assume that $A=\left\{a_{1},a_{2},\ldots ,a_{n}\right\}.$ We will consider two cases: the case of R>1, and the case of 0<R<1. If R>1, then $\mu {\left(a_{i}\right)}^{R}\leq \mu (a_{i}),$ for i=1,2,…,n, hence $\sum_{i=1}^{n}\mu {\left(a_{i}\right)}^{R}\leq \sum_{i=1}^{n}\mu (a_{i})=1.$ This implies that ${\left[\sum_{i=1}^{n}\mu {\left(a_{i}\right)}^{R}\right]}^{\frac{1}{R}}\leq 1.$ Since $\frac{R}{R-1}>0$ for R>1, it follows that $H_{R}^{\mu }(A)=$ $\frac{R}{R-1}\left(1-{\left[\sum_{i=1}^{n}\mu {\left(a_{i}\right)}^{R}\right]}^{\frac{1}{R}}\right)\geq 0.$ On the other hand, for 0<R<1, it holds that $\mu {\left(a_{i}\right)}^{R}\geq \mu (a_{i}),$ for i=1,2,…,n, hence $\sum_{i=1}^{n}\mu {\left(a_{i}\right)}^{R}\geq \sum_{i=1}^{n}\mu (a_{i})=1.$ It follows that ${\left[\sum_{i=1}^{n}\mu {\left(a_{i}\right)}^{R}\right]}^{\frac{1}{R}}\geq 1.$ Since $\frac{R}{R-1}<0$ for 0<R<1, we obtain $H_{R}^{\mu }(A)=\frac{R}{R-1}\left(1-{\left[\sum_{i=1}^{n}\mu {\left(a_{i}\right)}^{R}\right]}^{\frac{1}{R}}\right)\geq 0.$ □

**Example** **2.** *Let* X=[0,1], *and* a:X→[0,1] *be defined by* a(x)=x, x∈X. *Consider a fuzzy measurable space* (X,M), *where* $M=\left\{1_{X},\;0_{X},\;a,\;a^{⊥},\;a\cup a^{⊥},\;a\cap a^{⊥}\right\}.$ *Then it can be easily verified that the mappings* μ:M→[0,1], *and* υ:M→[0,1] *defined by the equalities* $\mu \left(1_{X}\right)=\mu (a\cup a^{⊥})=1,$ $\mu \left(0_{X}\right)=\mu (a\cap a^{⊥})=0,$ $\mu (a)=\mu (a^{⊥})=\frac{1}{2},$ $\upsilon \left(1_{X}\right)=\upsilon (a\cup a^{⊥})=1,$ $\upsilon \left(0_{X}\right)=\upsilon (a\cap a^{⊥})=0,$ $\upsilon (a)=\frac{1}{3},$ $\upsilon (a^{⊥})=\frac{2}{3},$ *are fuzzy P-measures and the systems* (X,M,μ), (X,M,υ) *are fuzzy probability spaces. The sets* $A=\left\{a,a^{⊥}\right\},$ $B=\left\{a\cup a^{⊥}\right\},$ $C=\left\{1_{X}\right\}$ *are fuzzy partitions of* (X,M,μ), *and* (X,M,υ) *such that* C≺B≺A. *We can calculate their R-norm entropy. Evidently,* $H_{R}^{\mu }(B)=H_{R}^{\mu }(C)=H_{R}^{\upsilon }(B)=H_{R}^{\upsilon }(C)=0;$ *in accordance with the natural requirement, experiments resulting in a certain event have zero R-norm entropy. Furthermore, we have:*

$$H_{R}^{\mu }(A)=\frac{R}{R-1}\left(1-{\left[{\left(\frac{1}{2}\right)}^{R}+{\left(\frac{1}{2}\right)}^{R}\right]}^{\frac{1}{R}}\right)=\frac{R}{R-1}\left(1-2^{\frac{1-R}{R}}\right),$$

$$H_{R}^{\upsilon }(A)=\frac{R}{R-1}\left(1-{\left[{\left(\frac{1}{3}\right)}^{R}+{\left(\frac{2}{3}\right)}^{R}\right]}^{\frac{1}{R}}\right).$$

*If we put* $R=\frac{1}{2},$ *then* $H_{R}^{\mu }(A)=1,$ $H_{R}^{\upsilon }(A)\dot{=}0.9428;$ *for* $R=\frac{1}{3},$ *we have* $H_{R}^{\mu }(A)=1.5,$ $H_{R}^{\upsilon }(A)\dot{=}1.4236;$ *for* R=2, *we have* $H_{R}^{\mu }(A)\dot{=}0.58579,$ *and* $H_{R}^{\upsilon }(A)\dot{=}0.50928.$

**Definition** **7.** *Let* $A=\left\{a_{1},a_{2},\ldots ,a_{n}\right\},$ *and* $B=\left\{b_{1},b_{2},\ldots ,b_{m}\right\}$ *be two fuzzy partitions of a fuzzy probability space* (X,M,μ). *Then the conditional R-norm entropy of A given B with respect to* μ *is defined, for a positive real number R not equal to* 1*, by the formula:*

(5) $$H_{R}^{\mu }(A/B)=\frac{R}{R-1}\left({\left[\sum_{j=1}^{m}\mu {\left(b_{j}\right)}^{R}\right]}^{\frac{1}{R}}-{\left[\sum_{j=1}^{m}\sum_{i=1}^{n}\mu {\left(a_{i}\cap b_{j}\right)}^{R}\right]}^{\frac{1}{R}}\right).$$

**Remark** **2.** *Let A be a fuzzy partition of a given fuzzy probability space* (X,M,μ). *Evidently, if we put* B={b}, *where* b∈M *is a W-universum, then* $H_{R}^{\mu }(A/B)=H_{R}^{\mu }(A).$

The following theorem shows the consistency of the conditional *R*-norm entropy $H_{R}^{\mu }(A/B),$ in the case of the limit of R going to 1, with the conditional Shannon entropy $H^{\mu }(A/B)$ defined by the formula (2), up to a positive multiplicative constant.

**Theorem** **2.** *Let* $A=\left\{a_{1},a_{2},\ldots ,a_{n}\right\},$ *and* $B=\left\{b_{1},b_{2},\ldots ,b_{m}\right\}$ *be two fuzzy partitions of a given fuzzy probability space* (X,M,μ). *Then* $\underset{R\to 1}{\lim}H_{R}^{\mu }(A/B)=c\cdot H^{\mu }(A/B),$ *where* $c=\frac{1}{\log e},$ *and* $H^{\mu }(A/B)=$ $-\sum_{i=1}^{n}\sum_{j=1}^{m}\mu (a_{i}\cap b_{j})\cdot \log\frac{\mu (a_{i}\cap b_{j})}{\mu (b_{j})}.$

**Proof.**  In the proof, we use L’Hôpital’s rule $\underset{R\to a}{\lim}\frac{f(R)}{g(R)}=\underset{R\to a}{\lim}\frac{f^{\prime }(R)}{g^{\prime }(R)},$ where in this case a=1. For every R∈(0,1)∪(1,∞), we can write:

$$H_{R}^{\mu }(A/B)=\frac{1}{1-\frac{1}{R}}\left({\left[\sum_{j=1}^{m}\mu {\left(b_{j}\right)}^{R}\right]}^{\frac{1}{R}}-{\left[\sum_{j=1}^{m}\sum_{i=1}^{n}\mu {\left(a_{i}\cap b_{j}\right)}^{R}\right]}^{\frac{1}{R}}\right)=\frac{f(R)}{g(R)},$$

where f,g are continuous functions defined for R∈(0,∞) in the following way:

$$f(R)={\left[\sum_{j=1}^{m}\mu {\left(b_{j}\right)}^{R}\right]}^{\frac{1}{R}}-{\left[\sum_{j=1}^{m}\sum_{i=1}^{n}\mu {\left(a_{i}\cap b_{j}\right)}^{R}\right]}^{\frac{1}{R}},\;g(R)=1-\frac{1}{R}.$$

By continuity of the function g, we get $\underset{R\to 1}{\lim}g(R)=g(1)=0.$ Furthermore, by continuity of the function f, and by the property (P4) of fuzzy P-measure μ, we get $\underset{R\to 1}{\lim}f(R)=f(1)=\sum_{j=1}^{m}\mu (b_{j})-\sum_{j=1}^{m}\sum_{i=1}^{n}\mu (a_{i}\cap b_{j})=1-\sum_{j=1}^{m}\mu \left(\left(\cup_{i=1}^{n}a_{i}\right)\cap b_{j}\right)=1-\sum_{j=1}^{m}\mu \left(b_{j}\right)=1-1=0.$ Using L’Hôpital’s rule, this implies:

$$\underset{R\to 1}{\lim}H_{R}^{\mu }(A/B)=\frac{\underset{R\to 1}{\lim}f^{\prime }(R)}{\underset{R\to 1}{\lim}g^{\prime }(R)}$$

under the assumption that the right-hand side exists. To find the derivative of the function f(R) we use the identity $b^{\alpha }=e^{\alpha \ln b}.$ Let us calculate:

$$\frac{d}{dR}f(R)=e^{\frac{1}{R}\ln\sum_{j=1}^{m}\mu {\left(b_{j}\right)}^{R}}\cdot \left(-\frac{1}{R^{2}}\cdot \ln\sum_{j=1}^{m}\mu {\left(b_{j}\right)}^{R}+\frac{1}{R}\cdot \frac{1}{\sum_{j=1}^{m}\mu {\left(b_{j}\right)}^{R}}\sum_{j=1}^{m}\mu {\left(b_{j}\right)}^{R}\cdot \ln\mu (b_{j})\right)\;-e^{\frac{1}{R}\ln\sum_{i=1}^{n}\sum_{j=1}^{m}\mu {\left(a_{i}\cap b_{j}\right)}^{R}}\left(-\frac{1}{R^{2}}\cdot \ln\sum_{i=1}^{n}\sum_{j=1}^{m}\mu {\left(a_{i}\cap b_{j}\right)}^{R}+\frac{1}{R}\cdot \frac{1}{\sum_{i=1}^{n}\sum_{j=1}^{m}\mu {\left(a_{i}\cap b_{j}\right)}^{R}}\sum_{i=1}^{n}\sum_{j=1}^{m}\mu {\left(a_{i}\cap b_{j}\right)}^{R}\cdot \ln\mu (a_{i}\cap b_{j})\right).$$

Since $\underset{R\to 1}{\lim}g^{\prime }(R)=\underset{R\to 1}{\lim}\frac{1}{R^{2}}=1,$ we obtain:

$$\underset{R\to 1}{\lim}H_{R}^{\mu }(A/B)=\underset{R\to 1}{\lim}f^{\prime }(R)=\sum_{j=1}^{m}\mu (b_{j})\cdot \ln\mu (b_{j})-\sum_{i=1}^{n}\sum_{j=1}^{m}\mu (a_{i}\cap b_{j})\cdot \ln\mu (a_{i}\cap b_{j})\;=\sum_{j=1}^{m}\sum_{i=1}^{n}\mu (a_{i}\cap b_{j})\cdot \ln\mu (b_{j})-\sum_{i=1}^{n}\sum_{j=1}^{m}\mu (a_{i}\cap b_{j})\cdot \ln\mu (a_{i}\cap b_{j})\;=-\sum_{i=1}^{n}\sum_{j=1}^{m}\mu (a_{i}\cap b_{j})\cdot \left(\frac{\log\mu (a_{i}\cap b_{j})}{\log e}-\frac{\log\mu (b_{j})}{\log e}\right)=-\frac{1}{\log e}\sum_{i=1}^{n}\sum_{j=1}^{m}\mu (a_{i}\cap b_{j})\cdot \log\frac{\mu (a_{i}\cap b_{j})}{\mu (b_{j})}.\;□$$

**Theorem** **3.** *Let* $A=\left\{a_{1},a_{2},\ldots ,a_{n}\right\}$ *be a fuzzy partition of a fuzzy probability space* (X,M,μ). *Then* $\underset{R\to 1}{\lim}H_{R}^{\mu }(A)=c\cdot H^{\mu }(A),$ *where* $H^{\mu }(A)=-\sum_{i=1}^{n}\mu (a_{i})\cdot \log\mu (a_{i}),$ *and* $c=\frac{1}{\log e}.$

**Proof.**  The claim is a direct consequence of the previous theorem; it suffices to put $B=\left\{1_{X}\right\}.$ □

In the following, the properties of the *R*-norm entropy of fuzzy partitions are discussed.

**Theorem** **4.** *For arbitrary fuzzy partitions* A,B *and* C *of a fuzzy probability space* (X,M,μ), *it holds that:*

(6) $$H_{R}^{\mu }(A\vee B/C)=H_{R}^{\mu }(A/C)+H_{R}^{\mu }(B/A\vee C).$$

**Proof.**  Suppose that $A=\left\{a_{1},a_{2},\ldots ,a_{n}\right\},$ $B=\left\{b_{1},b_{2},\ldots ,b_{m}\right\},$ $C=\left\{c_{1},c_{2},\ldots ,c_{r}\right\}.$ Let us calculate:

$$H_{R}^{\mu }(A\vee B/C)=\frac{R}{R-1}\left({\left[\sum_{k=1}^{r}\mu {\left(c_{k}\right)}^{R}\right]}^{\frac{1}{R}}-{\left[\sum_{i=1}^{n}\sum_{j=1}^{m}\sum_{k=1}^{r}\mu {\left(a_{i}\cap b_{j}\cap c_{k}\right)}^{R}\right]}^{\frac{1}{R}}\right)\;=\frac{R}{R-1}\left({\left[\sum_{k=1}^{r}\mu {\left(c_{k}\right)}^{R}\right]}^{\frac{1}{R}}-{\left[\sum_{i=1}^{n}\sum_{k=1}^{r}\mu {\left(a_{i}\cap c_{k}\right)}^{R}\right]}^{\frac{1}{R}}\right)\;+\frac{R}{R-1}\left({\left[\sum_{i=1}^{n}\sum_{k=1}^{r}\mu {\left(a_{i}\cap c_{k}\right)}^{R}\right]}^{\frac{1}{R}}-{\left[\sum_{i=1}^{n}\sum_{j=1}^{m}\sum_{k=1}^{r}\mu {\left(a_{i}\cap b_{j}\cap c_{k}\right)}^{R}\right]}^{\frac{1}{R}}\right)\;=H_{R}^{\mu }(A/C)+H_{R}^{\mu }(B/A\vee C).\;□$$

**Theorem** **5.** *For arbitrary fuzzy partitions* A,B *of a fuzzy probability space* (X,M,μ), *it holds that:*

(7) $$H_{R}^{\mu }(A\vee B)=H_{R}^{\mu }(A)+H_{R}^{\mu }(B/A).$$

**Proof.**  The claim is a direct consequence of the previous theorem; it suffices to put $C=\left\{1_{X}\right\}.$ □

In the following theorem, using the notion of conditional *R*-norm entropy of fuzzy partitions, chain rules for the *R*-norm entropy of fuzzy partitions are established.

**Theorem** **6.** *Let* $A_{1},A_{2},\ldots ,A_{n}$ *and C be fuzzy partitions of a fuzzy probability space* (X,M,μ). *Then, for* n=2,3,…, *the following equalities hold:*

*(i)*  $H_{R}^{\mu }(A_{1}\vee A_{2}\vee \ldots \vee A_{n})=H_{R}^{\mu }(A_{1})+\sum_{i=2}^{n}H_{R}^{\mu }(A_{i}/\vee_{k=1}^{i-1}A_{k});$
*(ii)*  $H_{R}^{\mu }(\vee_{i=1}^{n}A_{i}/C)=H_{R}^{\mu }(A_{1}/C)+\sum_{i=2}^{n}H_{R}^{\mu }(A_{i}/(\vee_{k=1}^{i-1}A_{k})\vee C).$

**Proof.**  The proof can be done using mathematical induction and Theorems 4 and 5. □

In the following we prove that the *R*-norm entropy $H_{R}^{\mu }(A)$ is a concave function on the class of all fuzzy P-measures on a given fuzzy measurable space (X,M).

**Proposition** **1.** *Let* μ, υ *be two fuzzy P-measures on a given fuzzy measurable space* (X,M). *Then, for every real number* λ∈[0,1], *the map* λμ+(1−λ)υ:X→[0,1] *is a fuzzy P-measure on* (X,M).

**Proof.**  It is straightforward. □

**Theorem** **7.** *Let A be a fuzzy partition of fuzzy probability spaces* (X,M,μ), (X,M,υ). *Then, for every real number* λ∈[0,1], *this inequality holds:*

$$\lambda H_{R}^{\mu }(A)+(1-\lambda )H_{R}^{\upsilon }(A)\leq H_{R}^{\lambda \mu +(1-\lambda )\upsilon }(A).$$

**Proof.**  Let $A=\left\{a_{1},a_{2},\ldots ,a_{n}\right\},$ and λ∈[0,1]. Putting $x_{i}=\lambda \mu (a_{i}),$ and $y_{i}=(1-\lambda )\upsilon (a_{i}),$ for i=1,2,…,n, in the Minkowski inequality, we obtain for R>1:

$$\lambda {\left[\sum_{i=1}^{n}\mu {\left(a_{i}\right)}^{R}\right]}^{\frac{1}{R}}+(1-\lambda ){\left[\sum_{i=1}^{n}\upsilon {\left(a_{i}\right)}^{R}\right]}^{\frac{1}{R}}\geq {\left[\sum_{i=1}^{n}{\left(\lambda \mu (a_{i})+(1-\lambda )\upsilon (a_{i})\right)}^{R}\right]}^{\frac{1}{R}},$$

and for 0<R<1:

$$\lambda {\left[\sum_{i=1}^{n}\mu {\left(a_{i}\right)}^{R}\right]}^{\frac{1}{R}}+(1-\lambda ){\left[\sum_{i=1}^{n}\upsilon {\left(a_{i}\right)}^{R}\right]}^{\frac{1}{R}}\leq {\left[\sum_{i=1}^{n}{\left(\lambda \mu (a_{i})+(1-\lambda )\upsilon (a_{i})\right)}^{R}\right]}^{\frac{1}{R}}.$$

This means that the function $\mu \mapsto {\left[\sum_{i=1}^{n}\mu {\left(a_{i}\right)}^{R}\right]}^{\frac{1}{R}}$ is convex in μ for R>1, and concave in μ for 0<R<1. Therefore, the function $\mu \mapsto 1-{\left[\sum_{i=1}^{n}\mu {\left(a_{i}\right)}^{R}\right]}^{\frac{1}{R}}$ is concave in μ for R>1, and convex in μ for 0<R<1. Evidently, $\frac{R}{R-1}>0$ for R>1, and $\frac{R}{R-1}<0$ for 0<R<1. According to definition of the *R*-norm entropy $H_{R}^{\mu }(A),$ we obtain that for every R∈(0,1)∪(1,∞), the *R*-norm entropy $\mu \mapsto H_{R}^{\mu }(A)$ is a concave function on the family of all fuzzy P-measures on a given fuzzy measurable space (X,M). Thus, for every λ∈[0,1], it holds that:

$$\lambda H_{R}^{\mu }(A)+(1-\lambda )H_{R}^{\upsilon }(A)\leq H_{R}^{\lambda \mu +(1-\lambda )\upsilon }(A).\;□$$

**Proposition** **2.** *Let* A,B *be fuzzy partitions of a fuzzy probability space* (X,M,μ) *such that* A≺B. *Then there exists a partition* $\left\{I_{1},I_{2},\ldots ,I_{n}\right\}$ *of the set* {1,2,…,m} *such that* $\mu (a_{i})=\sum_{j\in I_{i}}\mu (b_{j}),$ *for* i=1,2,…,n.

**Proof.**  By the assumption, for every b∈B there exists a∈A such that b≤a. Let us denote by $I_{i}$ the subset of the set {1,2,…,m} such that for every $j\in I_{i},$ it holds that $b_{j}\leq a_{i},$ i=1,2,…,n. Then the set $\left\{I_{1},I_{2},\ldots ,I_{n}\right\}$ is a partition of the set {1,2,…,m} and $\cup_{j\in I_{i}}b_{j}\leq a_{i},$ for i=1,2,…,n. By monotonicity of fuzzy P-measure μ, $\mu (a_{i})\geq \mu (\cup_{j\in I_{i}}b_{j})=\sum_{j\in I_{i}}\mu (b_{j}),$ for i=1,2,…,n. Summing over i=1,2,…,n, we get $\sum_{i=1}^{n}\mu (a_{i})\geq \sum_{i=1}^{n}\sum_{j\in I_{i}}\mu (b_{j})=\sum_{j=1}^{m}\mu (b_{j})=1.$ Since $\sum_{i=1}^{n}\mu (a_{i})=1,$ it follows that $\mu (a_{i})=\sum_{j\in I_{i}}\mu (b_{j}),$ for i=1,2,…,n. □

**Theorem** **8.** *Let* A,B,C *be fuzzy partitions of a fuzzy probability space* (X,M,μ) *such that* A≺B. *Then:*

*(i)*  $H_{R}^{\mu }(A)\leq H_{R}^{\mu }(B);$
*(ii)*  $H_{R}^{\mu }(A/C)\leq H_{R}^{\mu }(B/C).$

**Proof.**  (i) Assume that $A=\left\{a_{1},a_{2},\ldots ,a_{n}\right\},$ $B=\left\{b_{1},b_{2},\ldots ,b_{m}\right\},$ A≺B. According to Proposition 2 there exists a partition $\left\{I_{1},I_{2},\ldots ,I_{n}\right\}$ of the set {1,2,…,m} such that $\mu (a_{i})=\sum_{j\in I_{i}}\mu (b_{j}),$ for i=1,2,…,n. For the case of R>1, we obtain:

$$\mu {\left(a_{i}\right)}^{R}={\left(\sum_{j\in I_{i}}\mu (b_{j})\right)}^{R}\geq {\sum_{j\in I_{i}}\mu (b_{j})}^{R},\;\mathrm{for}\;i=1,2,\ldots ,n,$$

and consequently:

$$\sum_{i=1}^{n}\mu {\left(a_{i}\right)}^{R}\geq \sum_{j=1}^{m}\mu {\left(b_{j}\right)}^{R}.$$

Hence

$${\left[\sum_{i=1}^{n}\mu {\left(a_{i}\right)}^{R}\right]}^{\frac{1}{R}}\geq {\left[\sum_{j=1}^{m}\mu {\left(b_{j}\right)}^{R}\right]}^{\frac{1}{R}}.$$

Since $\frac{R}{R-1}>0$ for R>1, we conclude that:

$$H_{R}^{\mu }(A)=\frac{R}{R-1}\left(1-{\left[\sum_{i=1}^{n}\mu {\left(a_{i}\right)}^{R}\right]}^{\frac{1}{R}}\right)\leq \frac{R}{R-1}\left(1-{\left[\sum_{j=1}^{m}\mu {\left(b_{j}\right)}^{R}\right]}^{\frac{1}{R}}\right)=H_{R}^{\mu }(B).$$

For the case of 0<R<1, we get:

$$\mu {\left(a_{i}\right)}^{R}={\left(\sum_{j\in I_{i}}\mu (b_{j})\right)}^{R}\leq {\sum_{j\in I_{i}}\mu (b_{j})}^{R},\;\mathrm{for}\;i=1,2,\ldots ,n,$$

and consequently:

$$\sum_{i=1}^{n}\mu {\left(a_{i}\right)}^{R}\leq \sum_{j=1}^{m}\mu {\left(b_{j}\right)}^{R}.$$

Therefore:

$${\left[\sum_{i=1}^{n}\mu {\left(a_{i}\right)}^{R}\right]}^{\frac{1}{R}}\leq {\left[\sum_{j=1}^{m}\mu {\left(b_{j}\right)}^{R}\right]}^{\frac{1}{R}}.$$

Since $\frac{R}{R-1}<0$ for 0<R<1, we have:

$$H_{R}^{\mu }(A)=\frac{R}{R-1}\left(1-{\left[\sum_{i=1}^{n}\mu {\left(a_{i}\right)}^{R}\right]}^{\frac{1}{R}}\right)\leq \frac{R}{R-1}\left(1-{\left[\sum_{j=1}^{m}\mu {\left(b_{j}\right)}^{R}\right]}^{\frac{1}{R}}\right)=H_{R}^{\mu }(B).$$

(ii) By the assumption, for every b∈B there exists a∈A such that b≤a. Hence, for arbitrary element b∩c of fuzzy partition B∨C there exists a∩c∈A∨C such that b∩c≤a∩c. This means that A∨C≺B∨C. Therefore, we get:

$$H_{R}^{\mu }(A/C)=H_{R}^{\mu }(A\vee C)-H_{R}^{\mu }(C)\leq H_{R}^{\mu }(B\vee C)-H_{R}^{\mu }(C)=H_{R}^{\mu }(B/C).\;□$$

**Theorem** **9.** *Let* A,B *be statistically independent fuzzy partitions of a fuzzy probability space* (X,M,μ). *Then:*

$$H_{R}^{\mu }(A/B)=H_{R}^{\mu }(A)-\frac{R-1}{R}H_{R}^{\mu }(A)\cdot H_{R}^{\mu }(B).$$

**Proof.**  Let $A=\left\{a_{1},a_{2},\ldots ,a_{n}\right\},$ and $B=\left\{b_{1},b_{2},\ldots ,b_{m}\right\}.$ By the assumption, $\mu (a_{i}\cap b_{j})=\mu (a_{i})\cdot \mu (b_{j}),$ for i=1,2,…,n,j=1,2,…,m. Therefore we can write:

$$H_{R}^{\mu }(A/B)=\frac{R}{R-1}\left({\left[\sum_{j=1}^{m}\mu {\left(b_{j}\right)}^{R}\right]}^{\frac{1}{R}}-{\left[\sum_{j=1}^{m}\sum_{i=1}^{n}\mu {\left(a_{i}\cap b_{j}\right)}^{R}\right]}^{\frac{1}{R}}\right)\;=\frac{R}{R-1}\left({\left[\sum_{j=1}^{m}\mu {\left(b_{j}\right)}^{R}\right]}^{\frac{1}{R}}-{\left[\sum_{j=1}^{m}\mu {\left(b_{j}\right)}^{R}\right]}^{\frac{1}{R}}{\left[\sum_{i=1}^{n}\mu {\left(a_{i}\right)}^{R}\right]}^{\frac{1}{R}}\right)\;=\frac{R}{R-1}(1-{\left[\sum_{i=1}^{n}\mu {\left(a_{i}\right)}^{R}\right]}^{\frac{1}{R}}-1+{\left[\sum_{i=1}^{n}\mu {\left(a_{i}\right)}^{R}\right]}^{\frac{1}{R}}+{\left[\sum_{j=1}^{m}\mu {\left(b_{j}\right)}^{R}\right]}^{\frac{1}{R}}-{\left[\sum_{j=1}^{m}\mu {\left(b_{j}\right)}^{R}\right]}^{\frac{1}{R}}{\left[\sum_{i=1}^{n}\mu {\left(a_{i}\right)}^{R}\right]}^{\frac{1}{R}})\;=\frac{R}{R-1}\left(1-{\left[\sum_{i=1}^{n}\mu {\left(a_{i}\right)}^{R}\right]}^{\frac{1}{R}}\right)-\frac{R-1}{R}\left(\frac{R}{R-1}\left(1-{\left[\sum_{i=1}^{n}\mu {\left(a_{i}\right)}^{R}\right]}^{\frac{1}{R}}\right)\cdot \frac{R}{R-1}\left(1-{\left[\sum_{j=1}^{m}\mu {\left(b_{j}\right)}^{R}\right]}^{\frac{1}{R}}\right)\right)\;=H_{R}^{\mu }(A)-\frac{R-1}{R}H_{R}^{\mu }(A)\cdot H_{R}^{\mu }(B).\;□$$

In view of Theorems 5 and 9, the *R*-norm entropy does not have the property of additivity, but it satisfies the property that is called pseudo-additivity, as stated in the following theorem.

**Theorem** **10.** (Pseudo-additivity). *Let* A,B *be statistically independent fuzzy partitions of a fuzzy probability space* (X,M,μ). *Then:*

$$H_{R}^{\mu }(A\vee B)=H_{R}^{\mu }(A)+H_{R}^{\mu }(B)-\frac{R-1}{R}H_{R}^{\mu }(A)\cdot H_{R}^{\mu }(B).$$

**Proof.**  The result follows by combining Theorems 5 and 9. □

## 4. The *R*-Norm Divergence of Fuzzy P-Measures

In this part, the concept of the *R*-norm divergence of fuzzy P-measures is defined. In order to avoid expressions like $\frac{0}{0},$ we will use in this section the following simplification: for any fuzzy partition $A=\left\{a_{1},a_{2},\ldots ,a_{n}\right\}$ of a fuzzy probability space (X,M,μ), we assume that $\mu (a_{i})>$0, for i=1,2,…,n. Note that this is without loss of generality, because $\sum_{i=1}^{n}\mu (a_{i})=\sum_{i:\mu (a_{i})>0}\mu (a_{i})$. We will prove basic properties of this quantity. The results are illustrated with numerical examples.

**Definition** **8.** *Let* μ, υ *be two fuzzy P-measures on a fuzzy measurable space* (X,M), *and* $A=\left\{a_{1},a_{2},\ldots ,a_{n}\right\}$ *be a fuzzy partition of fuzzy probability spaces* (X,M,μ), (X,M,υ). *The R-norm divergence of fuzzy P-measures* μ,υ *with respect to* A *is defined, for a positive real number R not equal to* 1*, as the number:*

(8) $$D_{R}^{A}(\mu ∥\upsilon )=\frac{R}{R-1}\left({\left[\sum_{i=1}^{n}\mu {\left(a_{i}\right)}^{R}\upsilon {\left(a_{i}\right)}^{1-R}\right]}^{\frac{1}{R}}-1\right).$$

**Remark** **3.** *It is easy to see that, for any fuzzy partition* A *of a fuzzy probability space* (X,M,μ), *we have* $D_{R}^{A}(\mu ∥\mu )=0.$

The following theorem states that the *R*-norm divergence $D_{R}^{A}(\mu ∥\upsilon )$ is consistent, in the case of the limit of *R* going to 1, with the Kullback–Leibler divergence $D_{A}(\mu ∥\upsilon )$ defined by formula (3), up to a positive multiplicative constant.

**Theorem** **11.** *Let* $A=\left\{a_{1},a_{2},\ldots ,a_{n}\right\}$ *be a fuzzy partition of fuzzy probability spaces* (X,M,μ), (X,M,υ). *Then* $\underset{R\to 1}{\lim}D_{R}^{A}(\mu ∥\upsilon )=c\cdot D_{A}(\mu ∥\upsilon ),$ *where* $D_{A}(\mu ∥\upsilon )=\sum_{i=1}^{n}\mu (a_{i})\log\frac{\mu (a_{i})}{\upsilon (a_{i})},$ *and* $c=\frac{1}{\log e}.$

**Proof.**  For every R∈(0,1)∪(1,∞), we can write:

$$D_{R}^{A}(\mu ∥\upsilon )=\frac{1}{1-\frac{1}{R}}\left({\left[\sum_{i=1}^{n}\mu {\left(a_{i}\right)}^{R}\upsilon {\left(a_{i}\right)}^{1-R}\right]}^{\frac{1}{R}}-1\right)=\frac{f(R)}{g(R)},$$

where f,g are continuous functions defined for R∈(0,∞) by the formulas:

$$f(R)={\left[\sum_{i=1}^{n}\mu {\left(a_{i}\right)}^{R}\upsilon {\left(a_{i}\right)}^{1-R}\right]}^{\frac{1}{R}}-1,\;g(R)=1-\frac{1}{R}.$$

By continuity of the functions f,g, we get $\underset{R\to 1}{\lim}f(R)=f(1)=\sum_{i=1}^{n}\mu (a_{i})\upsilon {\left(a_{i}\right)}^{0}-1$ $=\sum_{i=1}^{n}\mu (a_{i})-1$=1−1=0, and $\underset{R\to 1}{\lim}g(R)=g(1)=0.$ Using L’Hôpital’s rule this implies that:

$$\underset{R\to 1}{\lim}D_{R}^{A}(\mu ∥\upsilon )=\frac{\underset{R\to 1}{\lim}f^{\prime }(R)}{\underset{R\to 1}{\lim}g^{\prime }(R)}$$

under the assumption that the right-hand side exists. Let us calculate the derivative of the function f(R):

$$\frac{d}{dR}f(R)=e^{\frac{1}{R}\ln\sum_{i=1}^{n}\mu {\left(a_{i}\right)}^{R}\upsilon {\left(a_{i}\right)}^{1-R}}\cdot (-\frac{1}{R^{2}}\cdot \ln\sum_{i=1}^{n}\mu {\left(a_{i}\right)}^{R}\upsilon {\left(a_{i}\right)}^{1-R}\;+\frac{1}{R}\cdot \frac{1}{\sum_{i=1}^{n}\mu {\left(a_{i}\right)}^{R}\upsilon {\left(a_{i}\right)}^{1-R}}\sum_{i=1}^{n}\left(-\mu {\left(a_{i}\right)}^{R}\upsilon {\left(a_{i}\right)}^{1-R}\cdot \ln\upsilon (a_{i})+\upsilon {\left(a_{i}\right)}^{1-R}\mu {\left(a_{i}\right)}^{R}\cdot \ln\mu (a_{i})\right)).$$

Since $\underset{R\to 1}{\lim}g^{\prime }(R)=\underset{R\to 1}{\lim}\frac{1}{R^{2}}=1,$ we get:

$$\underset{R\to 1}{\lim}D_{R}^{A}(\mu ∥\upsilon )=\underset{R\to 1}{\lim}f^{\prime }(R)=\sum_{i=1}^{n}\left(\mu (a_{i})\cdot \ln\mu (a_{i})-\mu (a_{i})\cdot \ln\upsilon (a_{i})\right)\;=\sum_{i=1}^{n}\left(\mu (a_{i})\cdot \frac{\log\mu (a_{i})}{\log e}-\mu (a_{i})\cdot \frac{\log\upsilon (a_{i})}{\log e}\right)\;=\frac{1}{\log e}\sum_{i=1}^{n}\mu (a_{i})\cdot \log\frac{\mu (a_{i})}{\upsilon (a_{i})}=\frac{1}{\log e}D_{A}(\mu ∥\upsilon ).\;□$$

**Remark** **4.** *Evidently, if the Kullback–Leibler divergence* $D_{A}(\mu ∥\upsilon )$ *is expressed in terms of a natural logarithm, then it is the limiting case of the R-norm divergence* $D_{R}^{A}(\mu ∥\upsilon )$ *for R going to 1.*

Let $A=\left\{a_{1},a_{2},\ldots ,a_{n}\right\}$ be a fuzzy partition of fuzzy probability spaces (X,M,μ), (X,M,υ). In [32], it has been shown that for the Kullback–Leibler divergence $D_{A}(\mu ∥\upsilon )$ it holds the Gibbs inequality $D_{A}(\mu ∥\upsilon )\geq 0$ with the equality if and only if $\mu (a_{i})=\upsilon (a_{i}),$ for i=1,2,…,n. This result allows us to interpret the Kullback–Leibler divergence $D_{A}(\mu ∥\upsilon )$ as a distance measure between two fuzzy P-measures (over the same fuzzy partition). In the following theorem, we present an analogy of this result for the case of the *R*-norm divergence.

**Theorem** **12.** *Let* $A=\left\{a_{1},a_{2},\ldots ,a_{n}\right\}$ *be a fuzzy partition of fuzzy probability spaces* (X,M,μ), (X,M,υ). *Then* $D_{R}^{A}(\mu ∥\upsilon )\geq 0$ *with the equality if and only if* $\mu (a_{i})=\upsilon (a_{i}),$ *for* i=1,2,…,n.

**Proof.**  We shall consider two cases: the case of R>1, and the case of 0<R<1.

Consider the case of R>1. The inequality follows from Jensen’s inequality for the function φ defined by $\varphi (x)=x^{1-R},$ for every x∈[0,∞), and putting $\alpha_{i}=\mu (a_{i}),$ $x_{i}=\frac{\upsilon (a_{i})}{\mu (a_{i})},$ for i=1,2,…,n. The assumption that R>1 implies 1−R<0, hence the function φ is convex. Therefore, by Jensen’s inequality we obtain:

(9) $$1={\left(\sum_{i=1}^{n}\nu (a_{i})\right)}^{1-R}={\left(\sum_{i=1}^{n}\mu (a_{i})\frac{\nu (a_{i})}{\mu (a_{i})}\right)}^{1-R}\leq {\sum_{i=1}^{n}\mu (a_{i})\left(\frac{\nu (a_{i})}{\mu (a_{i})}\right)}^{1-R}=\sum_{i=1}^{n}\mu {\left(a_{i}\right)}^{R}\nu {\left(a_{i}\right)}^{1-R},$$

and consequently:

$${\left[\sum_{i=1}^{n}\mu {\left(a_{i}\right)}^{R}\upsilon {\left(a_{i}\right)}^{1-R}\right]}^{\frac{1}{R}}\geq 1.$$

Since $\frac{R}{R-1}>0$ for R>1, it follows that:

$$D_{R}^{A}(\mu ∥\upsilon )=\frac{R}{R-1}\left({\left[\sum_{i=1}^{n}\mu {\left(a_{i}\right)}^{R}\upsilon {\left(a_{i}\right)}^{1-R}\right]}^{\frac{1}{R}}-1\right)\geq 0.$$

For 0<R<1, the function φ defined by $\varphi (x)=x^{1-R},$ for every x∈[0,∞), is concave. Hence, using the Jensen inequality, we obtain:

$$1={\left(\sum_{i=1}^{n}\nu (a_{i})\right)}^{1-R}={\left(\sum_{i=1}^{n}\mu (a_{i})\frac{\nu (a_{i})}{\mu (a_{i})}\right)}^{1-R}\geq {\sum_{i=1}^{n}\mu (a_{i})\left(\frac{\nu (a_{i})}{\mu (a_{i})}\right)}^{1-R}=\sum_{i=1}^{n}\mu {\left(a_{i}\right)}^{R}\nu {\left(a_{i}\right)}^{1-R},$$

and consequently:

$${\left[\sum_{i=1}^{n}\mu {\left(a_{i}\right)}^{R}\upsilon {\left(a_{i}\right)}^{1-R}\right]}^{\frac{1}{R}}\leq 1.$$

Since $\frac{R}{R-1}<0$ for 0<R<1, we conclude that:

$$D_{R}^{A}(\mu ∥\upsilon )=\frac{R}{R-1}\left({\left[\sum_{i=1}^{n}\mu {\left(a_{i}\right)}^{R}\upsilon {\left(a_{i}\right)}^{1-R}\right]}^{\frac{1}{R}}-1\right)\geq 0.$$

The equality in (9) holds if and only if $\frac{\nu (a_{i})}{\mu (a_{i})}$ is constant, for i=1,2,…,n, i.e., if and only if $\nu (a_{i})=c\mu (a_{i}),$ for i=1,2,…,n. Taking the sum over all i=1,2,…,n, we get $\sum_{i=1}^{n}\nu (a_{i})=$$c\sum_{i=1}^{n}\mu (a_{i}),$ which implies that c=1. Therefore, $\nu (a_{i})=\mu (a_{i}),$ for i=1,2,…,n. This means that $D_{R}^{A}(\mu ∥\upsilon )=0$ if and only if $\mu (a_{i})=\upsilon (a_{i}),$ for i=1,2,…,n. □

In the example that follows, it is shown that the equality $D_{R}^{A}(\mu ∥\upsilon )=D_{R}^{A}(\upsilon ∥\mu )$ is not necessarily true which means that the *R*-norm divergence $D_{R}^{A}(\mu ∥\upsilon )$ is not symmetrical. Therefore, it is not a metric in a true sense.

**Example** **3.** *Consider the fuzzy probability spaces* (X,M,μ), (X,M,υ) *defined in Example 2 and the fuzzy partition* $A=\left\{a,a^{⊥}\right\}$ *of* (X,M,μ), (X,M,υ). *Let us calculate the R-norm divergencies* $D_{R}^{A}(\mu ∥\upsilon ),$ *and* $D_{R}^{A}(\upsilon ∥\mu ):$

$$D_{R}^{A}(\mu ∥\upsilon )=\frac{R}{R-1}\left({\left[{\left(\frac{1}{2}\right)}^{R}{\left(\frac{1}{3}\right)}^{1-R}+{\left(\frac{1}{2}\right)}^{R}{\left(\frac{2}{3}\right)}^{1-R}\right]}^{\frac{1}{R}}-1\right),\;D_{R}^{A}(\upsilon ∥\mu )=\frac{R}{R-1}\left({\left[{\left(\frac{1}{3}\right)}^{R}{\left(\frac{1}{2}\right)}^{1-R}+{\left(\frac{2}{3}\right)}^{R}{\left(\frac{1}{2}\right)}^{1-R}\right]}^{\frac{1}{R}}-1\right).$$

*Put* R=2. *Elementary calculations show that* $D_{R}^{A}(\mu ∥\upsilon )\dot{=}0.1213,$ *and* $D_{R}^{A}(\upsilon ∥\mu )\dot{=}0.1082,$ *thus* $D_{2}^{A}(\mu ∥\upsilon )\neq D_{2}^{A}(\upsilon ∥\mu ).$ *For* $R=\frac{1}{3},$ *we have* $D_{R}^{A}(\mu ∥\upsilon )\dot{=}0.0188,$ *and* $D_{R}^{A}(\upsilon ∥\mu )$$\dot{=}0.01908,$ *i.e.,* $D_{1/3}^{A}(\mu ∥\upsilon )\neq D_{1/3}^{A}(\upsilon ∥\mu ).$ *This means that* $D_{R}^{A}(\mu ∥\upsilon )\neq D_{R}^{A}(\upsilon ∥\mu ),$ *in general.*

**Theorem** **13.** *Let* μ, υ *be two fuzzy P-measures on a fuzzy measurable space* (X,M), *and* $A=\left\{a_{1},a_{2},\ldots ,a_{n}\right\}$ *be a fuzzy partition of fuzzy probability spaces* (X,M,μ), (X,M,υ). *In addition, let* υ *be uniform over* A, *i.e.,* $\upsilon (a_{i})=\frac{1}{n},$ *for* i=1,2,…,n. *Then, it holds that:*

(10) $$H_{R}^{\mu }(A)=\frac{R}{R-1}\left(1-n^{\frac{1-R}{R}}\right)-n^{\frac{1-R}{R}}\cdot D_{R}^{A}(\mu ∥\upsilon ).$$

**Proof.**  Let us calculate:

$$D_{R}^{A}(\mu ∥\upsilon )=\frac{R}{R-1}\left({\left[\sum_{i=1}^{n}\mu {\left(a_{i}\right)}^{R}\upsilon {\left(a_{i}\right)}^{1-R}\right]}^{\frac{1}{R}}-1\right)\;=\frac{R}{R-1}\left({\left[\sum_{i=1}^{n}\mu {\left(a_{i}\right)}^{R}n^{R-1}\right]}^{\frac{1}{R}}-1\right)=\frac{R}{R-1}\cdot n^{\frac{R-1}{R}}{\left[\sum_{i=1}^{n}\mu {\left(a_{i}\right)}^{R}\right]}^{\frac{1}{R}}-\frac{R}{R-1}\;=-n^{\frac{R-1}{R}}\cdot \frac{R}{R-1}\left(1-{\left[\sum_{i=1}^{n}\mu {\left(a_{i}\right)}^{R}\right]}^{\frac{1}{R}}\right)+n^{\frac{R-1}{R}}\cdot \frac{R}{R-1}-\frac{R}{R-1}\;=-n^{\frac{R-1}{R}}\cdot H_{R}^{\mu }(A)+\frac{R}{R-1}\left(n^{\frac{R-1}{R}}-1\right).$$

It follows that:

$$H_{R}^{\mu }(A)=\frac{R}{R-1}\left(1-n^{\frac{1-R}{R}}\right)-n^{\frac{1-R}{R}}\cdot D_{R}^{A}(\mu ∥\upsilon ).\;□$$

**Example** **4.** *Consider the fuzzy P-measures* μ, υ *from Example 2 and the fuzzy partition* $A=\left\{a,a^{⊥}\right\}$ *of fuzzy probability spaces* (X,M,μ), (X,M,υ). *The fuzzy P-measure* μ *is uniform over A. Put* R=2. *Based on previous results, we have* $D_{R}^{A}(\upsilon ∥\mu )\dot{=}0.1082,$ *and* $H_{R}^{\upsilon }(A)\dot{=}0.50928.$ *Let us calculate:*

$$\frac{R}{R-1}\left(1-2^{\frac{1-R}{R}}\right)-2^{\frac{1-R}{R}}\cdot D_{R}^{A}(\upsilon ∥\mu )\dot{=}2\left(1-2^{-\frac{1}{2}}\right)-2^{-\frac{1}{2}}\cdot 0.1082\dot{=}0.50928.$$

Thus, the Equality (10) holds.

As a direct consequence of Theorems 12 and 13, we obtain the following property of the *R*-norm entropy of fuzzy partitions:

**Corollary** **1.** *For arbitrary fuzzy partition* $A=\left\{a_{1},a_{2},\ldots ,a_{n}\right\}$ *of a fuzzy probability space* (X,M,μ), *it holds that:*

$$H_{R}^{\mu }(A)\leq \frac{R}{R-1}\left(1-n^{\frac{1-R}{R}}\right)$$

*with the equality if and only if the fuzzy P-measure* μ *is uniform over A.*

**Theorem** **14.** *Let* $\mu_{1},$ $\mu_{2},$ υ *be fuzzy P-measures on a fuzzy measurable space* $(X,M,\mu_{1}),$ *and A be a fuzzy partition of fuzzy probability spaces* $(X,M,\mu_{2}),$ (X,M,υ). *Then, for every real number* λ∈[0,1], *it holds that:*

$$D_{R}^{A}(\lambda \mu_{1}+(1-\lambda )\mu_{2}∥\upsilon )\leq \lambda D_{R}^{A}(\mu_{1}∥\upsilon )+(1-\lambda )D_{R}^{A}(\mu_{2}∥\upsilon ).$$

**Proof.**  Assume that $A=\left\{a_{1},a_{2},\ldots ,a_{n}\right\},$ and λ∈[0,1]. Putting $x_{i}=\lambda \mu_{1}(a_{i})\upsilon {\left(a_{i}\right)}^{\frac{1-R}{R}},$ and $y_{i}=(1-\lambda )\mu_{2}(a_{i})\upsilon {\left(a_{i}\right)}^{\frac{1-R}{R}},$ i=1,2,…,n, in the Minkowski inequality, we get for R>1:

$${\left[\sum_{i=1}^{n}{\left(\lambda \mu_{1}(a_{i})+(1-\lambda )\mu_{2}(a_{i})\right)}^{R}\upsilon {\left(a_{i}\right)}^{1-R}\right]}^{\frac{1}{R}}={\left[\sum_{i=1}^{n}{\left((\lambda \mu_{1}(a_{i})+(1-\lambda )\mu_{2}(a_{i}))\upsilon {\left(a_{i}\right)}^{\frac{1-R}{R}}\right)}^{R}\right]}^{\frac{1}{R}}\;={\left[\sum_{i=1}^{n}{\left(\lambda \mu_{1}(a_{i})\upsilon {\left(a_{i}\right)}^{\frac{1-R}{R}}+(1-\lambda )\mu_{2}(a_{i})\upsilon {\left(a_{i}\right)}^{\frac{1-R}{R}}\right)}^{R}\right]}^{\frac{1}{R}}\;\leq {\left[\sum_{i=1}^{n}{\left(\lambda \mu_{1}(a_{i})\upsilon {\left(a_{i}\right)}^{\frac{1-R}{R}}\right)}^{R}\right]}^{\frac{1}{R}}+{\left[{\sum_{i=1}^{n}\left((1-\lambda )\mu_{2}(a_{i})\upsilon {\left(a_{i}\right)}^{\frac{1-R}{R}}\right)}^{R}\right]}^{\frac{1}{R}}\;=\lambda {\left[\sum_{i=1}^{n}\mu_{1}{\left(a_{i}\right)}^{R}\upsilon {\left(a_{i}\right)}^{1-R}\right]}^{\frac{1}{R}}+(1-\lambda ){\left[\sum_{i=1}^{n}\mu_{2}{\left(a_{i}\right)}^{R}\upsilon {\left(a_{i}\right)}^{1-R}\right]}^{\frac{1}{R}},$$

and for 0<R<1:

$${\left[\sum_{i=1}^{n}{\left(\lambda \mu_{1}(a_{i})+(1-\lambda )\mu_{2}(a_{i})\right)}^{R}\upsilon {\left(a_{i}\right)}^{1-R}\right]}^{\frac{1}{R}}\;\geq {\left[\sum_{i=1}^{n}{\left(\lambda \mu_{1}(a_{i})\upsilon {\left(a_{i}\right)}^{\frac{1-R}{R}}\right)}^{R}\right]}^{\frac{1}{R}}+{\left[{\sum_{i=1}^{n}\left((1-\lambda )\mu_{2}(a_{i})\upsilon {\left(a_{i}\right)}^{\frac{1-R}{R}}\right)}^{R}\right]}^{\frac{1}{R}}\;=\lambda {\left[\sum_{i=1}^{n}\mu_{1}{\left(a_{i}\right)}^{R}\upsilon {\left(a_{i}\right)}^{1-R}\right]}^{\frac{1}{R}}+(1-\lambda ){\left[\sum_{i=1}^{n}\mu_{2}{\left(a_{i}\right)}^{R}\upsilon {\left(a_{i}\right)}^{1-R}\right]}^{\frac{1}{R}}.$$

This means that the function $\mu \mapsto {\left[\sum_{i=1}^{n}\mu {\left(a_{i}\right)}^{R}\upsilon {\left(a_{i}\right)}^{1-R}\right]}^{\frac{1}{R}}$ is convex in μ for R>1, and concave in μ for 0<R<1. The same applies to the function $\mu \mapsto {\left[\sum_{i=1}^{n}\mu {\left(a_{i}\right)}^{R}\upsilon {\left(a_{i}\right)}^{1-R}\right]}^{\frac{1}{R}}-1.$ Since $\frac{R}{R-1}>0$ for R>1, and $\frac{R}{R-1}<0$ for 0<R<1, we conclude that the function $\mu \mapsto D_{R}^{A}(\mu ∥\upsilon )$ is convex on the family of all fuzzy P-measures on a given fuzzy measurable space (X,M). Thus, for every real number λ∈[0,1], it holds that:

$$D_{R}^{A}(\lambda \mu_{1}+(1-\lambda )\mu_{2}∥\upsilon )\leq \lambda D_{R}^{A}(\mu_{1}∥\upsilon )+(1-\lambda )D_{R}^{A}(\mu_{2}∥\upsilon ).\;□$$

**Theorem** **15.** *Let* $A=\left\{a_{1},a_{2},\ldots ,a_{n}\right\}$ *be any fuzzy partition of a fuzzy probability space* (X,M,μ). *Then:*

*(i)*  0<R<1 *implies* $D_{R}^{A}(\mu ∥\upsilon )\leq D_{A}(\mu ∥\upsilon );$
*(ii)*  R>1 *implies* $D_{R}^{A}(\mu ∥\upsilon )\geq D_{A}(\mu ∥\upsilon ),$
  *where*
  $$D_{A}(\mu ∥\upsilon )=\sum_{i=1}^{n}\mu (a_{i})\log\frac{\mu (a_{i})}{\upsilon (a_{i})}.$$

**Proof.**  In the proof we use the Jensen inequality for the concave function φ defined by φ(x)=logx, for x∈(0,∞), and putting $\alpha_{i}=\mu (a_{i}),$ $x_{i}={\left(\frac{\upsilon (a_{i})}{\mu (a_{i})}\right)}^{R-1},$ for i=1,2,…,n. Since the logarithm satisfies the condition logx≤x−1, for all real numbers x>0, we get:

(11) $$\log{\left[\sum_{i=1}^{n}\mu {\left(a_{i}\right)}^{R}\upsilon {\left(a_{i}\right)}^{1-R}\right]}^{\frac{1}{R}}\leq {\left[\sum_{i=1}^{n}\mu {\left(a_{i}\right)}^{R}\upsilon {\left(a_{i}\right)}^{1-R}\right]}^{\frac{1}{R}}-1.$$

Suppose that 0<R<1. Then $\frac{R}{R-1}<0$ and using the inequality (11) and the Jensen inequality, we can write:

$$D_{R}^{A}(\mu ∥\upsilon )=\frac{R}{R-1}\left({\left[\sum_{i=1}^{n}\mu {\left(a_{i}\right)}^{R}\upsilon {\left(a_{i}\right)}^{1-R}\right]}^{\frac{1}{R}}-1\right)\;\leq \frac{R}{R-1}\log{\left[\sum_{i=1}^{n}\mu {\left(a_{i}\right)}^{R}\upsilon {\left(a_{i}\right)}^{1-R}\right]}^{\frac{1}{R}}=\frac{1}{R-1}\log\sum_{i=1}^{n}\mu {\left(a_{i}\right)}^{R}\upsilon {\left(a_{i}\right)}^{1-R}\;=\frac{1}{R-1}\log\sum_{i=1}^{n}\mu (a_{i}){\left(\frac{\mu (a_{i})}{\upsilon (a_{i})}\right)}^{R-1}\leq \frac{1}{R-1}\sum_{i=1}^{n}\mu (a_{i})\log{\left(\frac{\mu (a_{i})}{\upsilon (a_{i})}\right)}^{R-1}\;=\sum_{i=1}^{n}\mu (a_{i})\log\frac{\mu (a_{i})}{\upsilon (a_{i})}=D_{A}(\mu ∥\upsilon ).$$

The case of R>1 can be obtained in the similar way. □

**Example** **5.** *Consider the fuzzy P-measures* μ, υ *from Example 2 and the fuzzy partition* $A=\left\{a,a^{⊥}\right\}$ *of fuzzy probability spaces* (X,M,μ), (X,M,υ). *Based on the results from Example 3, we have* $D_{2}^{A}(\mu ∥\upsilon )\dot{=}0.1213,$ $D_{2}^{A}(\upsilon ∥\mu )\dot{=}0.1082,$ $D_{1/3}^{A}(\mu ∥\upsilon )\dot{=}0.0188,$ $D_{1/3}^{A}(\upsilon ∥\mu )\dot{=}0.01908.$ *By simple calculation we get that* $D_{A}(\mu ∥\upsilon )\dot{=}0.084963,$ *and* $D_{A}(\upsilon ∥\mu )\dot{=}0.081703.$ *Thus, for* $R=\frac{1}{3}$ *we have* $D_{R}^{A}(\mu ∥\upsilon )\leq D_{A}(\mu ∥\upsilon ),$ $D_{R}^{A}(\upsilon ∥\mu )\leq D_{A}(\upsilon ∥\mu );$ *and for* R=2 *we have* $D_{R}^{A}(\mu ∥\upsilon )\geq D_{A}(\mu ∥\upsilon ),$ *and* $D_{R}^{A}(\upsilon ∥\mu )\geq D_{A}(\upsilon ∥\mu ),$ *which is consistent with the statement in the previous theorem.*

We conclude our contribution with the formulation of a chain rule for the *R*-norm divergence in the fuzzy case. First, we define the conditional version of the *R*-norm divergence of fuzzy P-measures.

**Definition** **9.** *Let* $A=\left\{a_{1},a_{2},\ldots ,a_{n}\right\},$ $B=\left\{b_{1},b_{2},\ldots ,b_{m}\right\}$ *be two fuzzy partitions of fuzzy probability spaces* (X,M,μ), (X,M,υ). *Then, we define the conditional divergence of fuzzy P-measures* μ, υ *with respect to B assuming a realization of A, for a positive real number R not equal to* 1*, as the number:*

$$D_{R}^{B/A}(\mu ∥\upsilon )=\frac{R}{R-1}\left({\left[\sum_{i=1}^{n}\sum_{j=1}^{m}\mu {\left(a_{i}\cap b_{j}\right)}^{R}\upsilon {\left(a_{i}\cap b_{j}\right)}^{1-R}\right]}^{\frac{1}{R}}-{\left[\sum_{i=1}^{n}\mu {\left(a_{i}\right)}^{R}\upsilon {\left(a_{i}\right)}^{1-R}\right]}^{\frac{1}{R}}\right).$$

**Theorem** **16.** *Let* A,B *be two fuzzy partitions of fuzzy probability spaces* (X,M,μ), (X,M,υ). *Then*

$$D_{R}^{A\vee B}(\mu ∥\upsilon )=D_{R}^{A}(\mu ∥\upsilon )+D_{R}^{B/A}(\mu ∥\upsilon ).$$

**Proof.**  Assume that $A=\left\{a_{1},a_{2},\ldots ,a_{n}\right\},$ $B=\left\{b_{1},b_{2},\ldots ,b_{m}\right\}.$ Then we have:

$$D_{R}^{A}(\mu ∥\upsilon )+D_{R}^{B/A}(\mu ∥\upsilon )=\frac{R}{R-1}\left({\left[\sum_{i=1}^{n}\mu {\left(a_{i}\right)}^{R}\upsilon {\left(a_{i}\right)}^{1-R}\right]}^{\frac{1}{R}}-1\right)\;+\frac{R}{R-1}\left({\left[\sum_{i=1}^{n}\sum_{j=1}^{m}\mu {\left(a_{i}\cap b_{j}\right)}^{R}\upsilon {\left(a_{i}\cap b_{j}\right)}^{1-R}\right]}^{\frac{1}{R}}-{\left[\sum_{i=1}^{n}\mu {\left(a_{i}\right)}^{R}\upsilon {\left(a_{i}\right)}^{1-R}\right]}^{\frac{1}{R}}\right)\;=\frac{R}{R-1}\left({\left[\sum_{i=1}^{n}\sum_{j=1}^{m}\mu {\left(a_{i}\cap b_{j}\right)}^{R}\upsilon {\left(a_{i}\cap b_{j}\right)}^{1-R}\right]}^{\frac{1}{R}}-1\right)=D_{R}^{A\vee B}(\mu ∥\upsilon ).\;□$$

## 5. Conclusions

In this article, we have extended the study of entropy measures and distance measures in the fuzzy case. Our goal was to introduce the concepts of *R*-norm entropy and *R*-norm divergence for the case of fuzzy probability spaces and to derive basic properties of these measures. Our results are presented in Section 3 and Section 4.

In Section 3, we have defined the *R*-norm entropy and conditional *R*-norm entropy of fuzzy partitions of a given fuzzy probability space and have examined the properties of the proposed entropy measures. In particular, it has been shown that the *R*-norm entropy of fuzzy partitions does not have the property of additivity, but it satisfies the property called pseudo-additivity, as stated in Theorem 10. In Theorem 6, chain rules for the *R*-norm entropy of fuzzy partitions are provided. Moreover, it was shown that the Shannon entropy and the conditional Shannon entropy of fuzzy partitions can be derived from the *R*-norm entropy and conditional *R*-norm entropy of fuzzy partitions, respectively, as the limiting cases for R→1.

In Section 4, the concept of *R*-norm divergence of fuzzy P-measures was introduced and the properties of this quantity have been proven. Specifically, it was shown that the Kullback–Leibler divergence defined and studied in [32] can be derived from the *R*-norm divergence of fuzzy P-measures, as the limiting case for R→1. The result of Theorem 12 allows us to interpret the *R*-norm divergence as a distance measure between two fuzzy P-measures. Theorem 13 provides a relationship between the *R*-norm divergence and the *R*-norm entropy of fuzzy partitions; Theorem 15 provides a relationship between the *R*-norm divergence and the Kullback–Leibler divergence of fuzzy P-measures. In addition, the concavity of *R*-norm entropy (Theorem 7) and convexity of *R*-norm divergence (Theorem 14) have been demonstrated. Finally, using the suggested concept of conditional *R*-norm divergence of fuzzy P-measures, the chain rule for the *R*-norm divergence of fuzzy P-measures was established. 

In the proofs, the Jensen inequality, L’Hôpital’s rule, and the Minkowski inequality were used. The results presented in Section 3 and Section 4 are illustrated with numerical examples.
